# Genome sequences of four endophytic Bacillaceae from wild lettuce

**DOI:** 10.1128/mra.00274-25

**Published:** 2025-09-12

**Authors:** O. Joshua Oyekanmi, David A. Baltrus, A. Elizabeth Arnold

**Affiliations:** 1 School of Plant Sciences, University of Arizona 124485, Tucson, Arizona, USA; 2 Pure and Applied Biology, College of Agriculture, Engineering and Science (COAES), Bowen University 683778, Iwo, Osun, Nigeria; 3 Ecosystem Genomics Graduate Interdisciplinary Program, University of Arizona 8041, Tucson, Arizona, USA; 4 Bio5 Institute, University of Arizona 124486, Tucson, Arizona, USA; 5 School of Animal and Comparative Biomedical Sciences, University of Arizona 737151, Tucson, Arizona, USA; 6 Department of Ecology and Evolutionary Biology, University of Arizona 164486 , Tucson, Arizona, USA; 7 Center for Agroecosystem Research in the Desert, University of Arizona 8041, Tucson, Arizona, USA; University of Strathclyde, Glasgow, United Kingdom

**Keywords:** genome analysis, endophytes, Bacillaceae, wild lettuce

## Abstract

Endophytic bacteria were isolated from roots and stems of prickly lettuce (*Lactuca serriola* L.), a wild relative of cultivated lettuce (*Lactuca sativa* L.) in Tucson, Arizona, USA. Here, we report draft genome sequences of four strains: *Priestia megaterium* SY0032, *Bacillus velezensis* SY1154, *Bacillus inaquosorum* SY1167, and *Bacillus subtilis* SY1483, assembled using reads arising from Oxford Nanopore 10.4 flow cells.

## ANNOUNCEMENT

Wild relatives of crops harbor diverse microbiomes that may enhance nutrition and resilience under environmental stress; however, domestication and intensive agricultural practices often reduce microbial diversity (1, 2). To identify beneficial endophytes for potential applications in sustainable agriculture (3), we sequenced four bacterial strains isolated from prickly lettuce, a wild relative of cultivated lettuce.

In October 2014, we collected naturally occurring, mature *Lactuca serriola* in sites with limited water, poor soil, and high heat stress in Tucson, AZ. Roots were excavated to a depth of 12 cm and gently shaken to remove soil. Stems were collected intact. All collected tissues were transported on ice to the laboratory and stored at 4°C for 24–72 hours until processing. Root and stem segments (each ca. 2 cm long) were surface-sterilized with ethanol (95%, 30 s), sodium hypochlorite (0.5%, 2 min), and ethanol (70%, 2 min) (4, 5). Segments were placed on lysogeny broth (LB) agar (per 1 L = 10 g tryptone, 5 g yeast extract, 10 g NaCl, pH 7.5 + with 15 g agar). Pure cultures that emerged from cut ends of the tissue were sub-cultured on LB agar and incubated for 2 days at 37°C (6). Single colonies of pure cultures were identified morphologically, confirmed by PCR and 16S rDNA sequencing (7, 8), and preserved in 80% glycerol at −80°C (9) as vouchers by the University of Arizona Gilbertson Mycological Herbarium, with accession numbers corresponding to strain names (10).

Strains SY0032, SY1154, SY1167, and SY1483 were revived in October 2024 by streaking onto LB agar with 50 mM NaCl. Single colonies arising from this subculture were picked to LB broth and grown overnight at 27°C with shaking at 220 rpm in LB broth. Genomic DNA was extracted from cultures with the Promega Wizard Genomic DNA Purification Kit (Promega, USA), with the addition of lysozyme as per the manual, but without additional RNase treatment and intentional shearing or size selection of DNA.

Genomic DNA was sequenced using Oxford Nanopore Technologies by Plasmidsaurus (USA), via their standard pipeline (11). Libraries were built with the Ligation Sequencing Kit V14 (enzyme E8.2.1) and sequenced on R10.4.1 flow cells (12). Default parameters were used for all software, unless otherwise specified. Guppy v6.0 was used for base calling in high accuracy mode, and reads <1,000 bp were discarded. Reads were assembled using Flye v2.9.1, with circularity of chromosomes and plasmids being called by this software (13). Annotations were performed by the Prokaryotic Genome Annotation Pipeline v6.9 at the National Center for Biotechnology Information (14). Species taxonomy (Table 1) was verified by comparing with reference genomes of type strains using EzBioCloud Average Nucleotide Identity (ANI) (15) and Genome Taxonomy Database Toolkit (16) using default settings. Assembly statistics indicate complete *Bacillus* genomes for SY1154, SY1167, and SY1483, while SY0032 yielded a draft genome of *Priestia megaterium* (Table 1). These genomic data provide insights into the functional potential of endophytic bacteria from prickly lettuce and lay a foundation for future functional and comparative analyses.

**TABLE 1:** Analysis and assembly metrics of genome sequences of four endophytic bacteria from the stems and roots of prickly lettuce

	SY0032	SY1154	SY1167	SY1483
Source material	Stem	Root	Root	Root
Field coordinates	32.2706°N, 110.8920°W	32.2850°N, 110.9436°W	32.2850°N, 110.9436°W	32.2547°N, 111.0061°W
Species taxonomy (Oxford Nanopore)	*P. megaterium*	*Bacillus velezensis*	*Bacillus inaquosorum*	*Bacillus subtilis*
ANI reference strain	GCF_011058275.1	GCF_002893845.1	GCF_030128945.2	GCA_039620435.1
Total DNA sequenced (bp)	489,202,313	633,092,171	466,701,786	1,324,074,023
Raw coverage (x)	89	156	109	321
Assembly size (bp)	5,472,439	4,044,968	4,245,627	4,117,990
Assembled coverage (x)	73	102	94	99
Contig N_50_ (bp)	5,025,108	4,040,000	4,237,664	4,117,989
Read N_50_ (bp)	45,966	89,498	51,138	87,391
Total number of reads (bp)	401,992	267,626	442,725	296,961
GC content (%)	37.9	46.5	44.0	43.8
Total number of contigs	12	2	1	1
Circularized contigs	7	1	1	1
Number of genes	5,825	3,986	4,302	4,273
Protein-coding	5,561	3,787	4,059	4,050
Biosample	SAMN44627542	SAMN44627543	SAMN44627544	SAMN44627545
GenBank accession	GCA_045888035.1	GCA_047302555.1	GCA_047302545.1	GCA_047302535.1
SRA accession	SRS23478179	SRS23478180	SRS23478181	SRS23478182

## Data Availability

Sequence data from these genomes are available at GenBank under BioProject accession number PRJNA1183431. Accession numbers for genome assemblies and SRA accession numbers for the Nanopore reads are shown in Table 1.

## References

[1] Alford BA. 2020. Microbial ecology of wild and domesticated chickpeas, Doctoral dissertation University of California, Davis

[2] Sun P, Yuan H, Pan J, Wu Z, Li W, Wang X, Kuang H, Chen J. 2024. A WOX homolog disrupted by a transposon led to the loss of spines and contributed to the domestication of lettuce. New Phytol 242:2857–2871. doi:10.1111/nph.19738 38584520

[3] Saikkonen K, Nissinen R, Helander M. 2020. Toward comprehensive plant microbiome research. Front Ecol Evol 8:61. doi:10.3389/fevo.2020.00061

[4] Garcia C, Pauli D, Plecki C, Alnasser H, Rozzi B, Calleja S, Arnold AE. 2024. The root endophytic microbiome shifts under drought in high-performing sorghum. Phytobiomes J 8:282–296. doi:10.1094/PBIOMES-09-23-0095-R

[5] Arnold AE, Maynard Z, Gilbert GS, Coley PD, Kursar TA. 2000. Are tropical fungal endophytes hyperdiverse? Ecol Lett 3:267–274. doi:10.1046/j.1461-0248.2000.00159.x

[6] Baltrus DA, Feng Q, Kvitko BH. 2022. Genome context influences evolutionary flexibility of nearly identical type III effectors in two phytopathogenic Pseudomonads. Front Microbiol 13:826365. doi:10.3389/fmicb.2022.826365 35250942 PMC8895235

[7] Lane DJ. 1991. 16S/23S rRNA sequencing, p 115–175. In Nucleic acid techniques in bacterial systematics

[8] Hoffman MT, Arnold AE. 2010. Diverse bacteria inhabit living hyphae of phylogenetically diverse fungal endophytes. Appl Environ Microbiol 76:4063–4075. doi:10.1128/AEM.02928-09 20435775 PMC2893488

[9] Colón Carrión NM. 2022. Plant-fungal symbioses: climate change, applications for plant production, and farmer education, Doctoral dissertation University of Arizona

[10] Huang Y-L, Bowman EA, Massimo NC, Garber NP, U’Ren JM, Sandberg DC, Arnold AE. 2018. Using collections data to infer biogeographic, environmental, and host structure in communities of endophytic fungi. Mycologia 110:47–62. doi:10.1080/00275514.2018.1442078 29863996

[11] Becker L, Steglich M, Fuchs S, Werner G, Nübel U. 2016. Comparison of six commercial kits to extract bacterial chromosome and plasmid DNA for MiSeq sequencing. Sci Rep 6:28063. doi:10.1038/srep28063 27312200 PMC4911584

[12] Baltrus DA, Clark M. 2020. Complete genome sequence of Pseudomonas coronafaciens pv. oryzae 1_6. Microbiol Resour Announc 9:e01564-19. doi:10.1128/MRA.01564-19 31948975 PMC6965593

[13] Kolmogorov M, Yuan J, Lin Y, Pevzner PA. 2019. Assembly of long, error-prone reads using repeat graphs. Nat Biotechnol 37:540–546. doi:10.1038/s41587-019-0072-8 30936562

[14] Tatusova T, DiCuccio M, Badretdin A, Chetvernin V, Nawrocki EP, Zaslavsky L, Lomsadze A, Pruitt KD, Borodovsky M, Ostell J. 2016. NCBI prokaryotic genome annotation pipeline. Nucleic Acids Res 44:6614–6624. doi:10.1093/nar/gkw569 27342282 PMC5001611

[15] Yoon SH, Ha SM, Lim J, Kwon S, Chun J. 2017. A large-scale evaluation of algorithms to calculate average nucleotide identity. Antonie Van Leeuwenhoek 110:1281–1286. doi:10.1007/s10482-017-0844-4 28204908

[16] Petkova M, Dimova M. 2024. Biological control of lettuce drop (Sclerotinia minor Jagger) using antagonistic Bacillus species. Appl Microbiol 4:1283–1293. doi:10.3390/applmicrobiol4030088

